# Ocular Syphilis in the Era of Resurgence: Clinical Characteristics and Diagnostic Challenges in a Single-Center Cohort

**DOI:** 10.3390/medicina62081473

**Published:** 2026-07-29

**Authors:** Şerife Altun Demircan, Gülşah Gelişigüzel, Gozde Orman, Esra Kaya Kılıç, Salih Cesur, Günay Tuncer Ertem, Sengül Üçer, Çiğdem Ataman Hatipoğlu, Sami Kınıklı

**Affiliations:** 1 Department of Infectious Diseases and Clinical Microbiology, Ankara Training and Research Hospital, University of Health Sciences, 06230 Ankara, Türkiye; 2 Department of Infectious Diseases and Clinical Microbiology, Ankara Etlik City Hospital, 06170 Ankara, Türkiye; 3 Department of Ophtalmology, Ankara Training and Research Hospital, University of Health Sciences, 06230 Ankara, Türkiye

**Keywords:** neurosyphilis, uveitis, sexually transmitted diseases, serology, HIV

## Abstract

*Background and Objectives*: The global resurgence of syphilis has been accompanied by an increasing number of ocular syphilis cases. Ocular syphilis can occur at any stage of infection and has diverse clinical manifestations, making diagnosis challenging. *Materials and Methods*: We retrospectively reviewed 21 adults diagnosed with ocular syphilis at a tertiary referral center between 2018 and 2023. Diagnosis was based on compatible ophthalmologic findings, together with positive treponemal serology. Demographic, clinical, ophthalmologic, laboratory, cerebrospinal fluid (CSF), imaging, and treatment data were analyzed. *Results*: A total of 21 patients (30 eyes) were included. The majority were male (87%), with a median age of 44 years, and bilateral involvement occurred in 43% of patients. Blurred vision and decreased visual acuity were the most common presenting symptoms (80%). Uveitis (68.4%), predominantly posterior, and optic neuritis (42.1%) were the most frequent ophthalmologic findings. Serum VDRL was negative in 5 of 21 patients despite positive treponemal serology. Among the 14 patients who underwent lumbar puncture, half had normal cerebrospinal fluid findings. All patients received neurosyphilis-directed antimicrobial therapy. The mean baseline BCVA was 0.38 ± 0.30 logMAR (range, 0–1), improving to 0.16 ± 0.28 logMAR (range, 0–1) at the final follow-up. The improvement in BCVA following treatment was statistically significant (*p* < 0.001). No eye showed deterioration in BCVA after treatment; visual acuity either improved or remained stable in all eyes. Additionally, all cases of uveitis and optic neuropathy achieved clinical remission during follow-up. *Conclusions*: Ocular syphilis remains a diagnostic challenge because of its heterogeneous clinical manifestations and the occurrence of negative serum VDRL results or normal cerebrospinal fluid findings in patients who underwent lumbar puncture. These findings highlight the limitations of relying solely on non-treponemal serology or cerebrospinal fluid analysis when evaluating patients with suspected ocular syphilis.

## 1. Introduction

Syphilis has shown a global resurgence since the early 2000s, particularly among men who have sex with men (MSM) [1]. A similar trend has also been observed in our country, where the number of reported syphilis cases in 2022 increased approximately sevenfold compared with 2006 [2]. This resurgence has been attributed to multiple interacting factors, including changes in sexual behavior, multiple sexual partners, illicit drug use, and other high-risk sexual behaviors, particularly among MSM [3,4]. This epidemiological resurgence has also been accompanied by an increasing recognition of rare but serious complications, particularly ocular syphilis.

Before the introduction of penicillin into clinical practice in 1943, syphilis was considered one of the leading causes of uveitis [5]. Although ocular syphilis is an uncommon manifestation of systemic syphilis, the number of reported cases has increased worldwide in recent years. However, the true incidence remains difficult to determine because ocular syphilis is likely underreported, while advances in ophthalmic imaging and diagnostic techniques have also improved case detection [6]. Beyond ocular involvement, untreated syphilis may also lead to serious systemic complications, including neurosyphilis, cardiovascular syphilis, and congenital syphilis [7]. Clinically, ocular syphilis may occur at any stage of infection. However, it has been reported most frequently during the secondary and latent stages of syphilis, although it may also occur during primary and tertiary disease. Ocular syphilis most commonly presents as posterior uveitis or panuveitis, although it may manifest as a broad spectrum of ocular findings, including interstitial keratitis, recurrent anterior uveitis, retinal vasculitis, and optic neuropathy [6,8]. Patients may present with a variety of ocular symptoms such as redness, pain, vision loss, floaters, photopsia, and photophobia [9].

*Treponema pallidum* disseminates hematogenously shortly after primary infection and may invade immune-privileged ocular tissues early in the course of infection [7]. Following ocular invasion, persistent spirochetal infection, together with the host immune-mediated inflammatory response, causes tissue injury involving the uvea, retina, choroid, optic nerve, and retinal vasculature, resulting in the broad spectrum of clinical manifestations observed in ocular syphilis [6].

The diagnosis of ocular syphilis remains challenging because of its highly variable clinical manifestations and its ability to mimic numerous inflammatory and infectious ocular diseases. Furthermore, no single clinical or laboratory finding is sufficient to establish or exclude the diagnosis, and serologic test results should always be interpreted in conjunction with compatible clinical and ophthalmologic findings. Importantly, non-treponemal serologic tests may be nonreactive in some patients with active ocular syphilis, and therefore a negative test should not delay further diagnostic evaluation when clinical suspicion is high [6,10]. 

Because *T. pallidum* may invade both the eye and the central nervous system early during infection, current guidelines recommend managing ocular syphilis using the same antimicrobial regimen as neurosyphilis, regardless of cerebrospinal fluid (CSF) findings [6]. Although lumbar puncture and CSF examination are not routinely required in patients without cranial nerve involvement or other neurological manifestations, they may still be performed on an individual basis as part of the diagnostic evaluation [11]. Notably, normal CSF findings do not exclude ocular syphilis or alter the recommended antimicrobial treatment approach. The role of adjunctive systemic corticosteroids remains controversial, and their clinical benefit has not been clearly established [6]. Although ocular syphilis is generally curable with appropriate antimicrobial therapy, delayed diagnosis and treatment have been associated with poorer visual outcomes, underscoring the importance of early recognition and prompt therapy [12]. 

In this study, we aimed to characterize the clinical presentation, diagnostic challenges, laboratory findings, and treatment of ocular syphilis in a single tertiary care center.

## 2. Methods

This single-center retrospective study included all patients aged ≥18 years who were hospitalized with a diagnosis of ocular syphilis in the Department of Infectious Diseases and Clinical Microbiology at Ankara Training and Research Hospital between 1 January 2018, and 31 December 2023. After the diagnosis of ocular syphilis, patients were admitted to the Department of Infectious Diseases and Clinical Microbiology for systemic treatment and further evaluation. Patients are primarily referred from the Department of Ophthalmology within our institution, with occasional referrals from external ophthalmology centers.

The diagnosis of ocular syphilis was based on compatible ophthalmologic findings (e.g., uveitis or optic neuritis), together with positive syphilis serology, including treponemal tests, such as the *Treponema pallidum* hemagglutination assay (TPHA) and enzyme-linked immunosorbent assay (ELISA), and/or the non-treponemal Venereal Disease Research Laboratory (VDRL) test. Alternative infectious and non-infectious causes of uveitis and optic neuritis were excluded through comprehensive clinical, laboratory, and imaging evaluations, as appropriate. The diagnosis was further supported by clinical improvement following standard antisyphilitic therapy. All available ocular findings were evaluated and confirmed by an ophthalmologist. During hospitalization and follow-up visits, comprehensive ophthalmologic examinations were performed. Best-corrected visual acuity was assessed using LogMAR charts. Intraocular pressure was measured with Goldmann applanation tonometry. Anterior segment evaluation was performed by slit-lamp biomicroscopy. Fundus examination was conducted after pupil dilation using a +90-diopter aspheric lens (Volk, Mentor, OH, USA). Uveitis classification was based on the Standardization of Uveitis Nomenclature (SUN) Working Group criteria [13]. Data regarding sexual orientation were not systematically available because this information was not routinely documented in medical records, and some patients did not disclose it. Therefore, only limited data were available for some patients living with Human Immunodeficiency Virus (HIV). Lumbar puncture (LP) was recommended for all patients to assess possible concomitant neurosyphilis; however, CSF analysis was performed only in patients who provided consent. CSF evaluation included cell count, protein and glucose levels, and CSF VDRL testing. HIV serology was assessed in all patients, and for individuals living with HIV, CD4 T-lymphocyte counts and HIV-RNA levels were recorded. The patients’ demographic characteristics, presenting symptoms, ophthalmologic findings, serology and CSF results, HIV co-infection status, and administered treatments were retrospectively obtained from medical records and the hospital information management system.

Ethical approval for the study was obtained from the Clinical Research Ethics Committee of Ankara Training and Research Hospital (Approval No. E-93471371-514.99-229966672, dated 22 November 2023). 

Statistical analyses were performed using IBM SPSS Statistics for Windows, version 26.0 (IBM Corp., Armonk, NY, USA). Continuous variables were summarized as mean ± standard deviation (SD) or median (minimum–maximum), as appropriate, whereas categorical variables were expressed as frequencies and percentages. The distribution of best-corrected visual acuity (BCVA) data was assessed for normality. Because BCVA values were not normally distributed, baseline and final BCVA measurements were compared using the Wilcoxon signed-rank test. Owing to the relatively small sample size, comparative analyses were limited and were considered hypothesis-generating. A two-sided *p* value < 0.05 was considered statistically significant.

## 3. Results

### 3.1. Patient Characteristics and Demographics

A total of 21 patients (30 eyes) with ocular syphilis were included in the study. The annual proportion of patients with ocular syphilis among all patients diagnosed with syphilis at our institution was 5.4% (6/112) in 2019, 7.0% (4/57) in 2020, 2.5% (2/79) in 2021, 3.6% (5/138) in 2022, and 3.9% (4/102) in 2023.

The majority of patients were male (87%), with a median age of 44 years (range: 22–78). Ocular involvement was bilateral in 43% of cases. Baseline demographic, clinical, serological, and treatment characteristics are summarized in Table 1.

### 3.2. Clinical and Ophthalmologic Features

The most common presenting symptoms were blurred vision and decreased visual acuity, reported by 80% of patients. Two patients had been referred from another center with an established diagnosis of ocular syphilis, and detailed ophthalmologic examination records at presentation were unavailable.

Ophthalmologic examination data were available for 19 patients. Among these, uveitis was detected in 13 patients (68.4%) and optic neuritis in eight patients (42.1%). Of the 13 patients with uveitis, nine (69.2%) had posterior uveitis, one had anterior uveitis, and three had panuveitis. Concomitant posterior uveitis and optic neuritis were observed in two patients (10.5%). 

Regarding syphilis stage, four patients were classified as having secondary syphilis based on compatible clinical findings. The remaining 17 patients had latent syphilis; however, differentiation between early latent and late latent syphilis was not possible because the timing of infection could not be reliably determined from the available clinical history.

**Figure 1:**
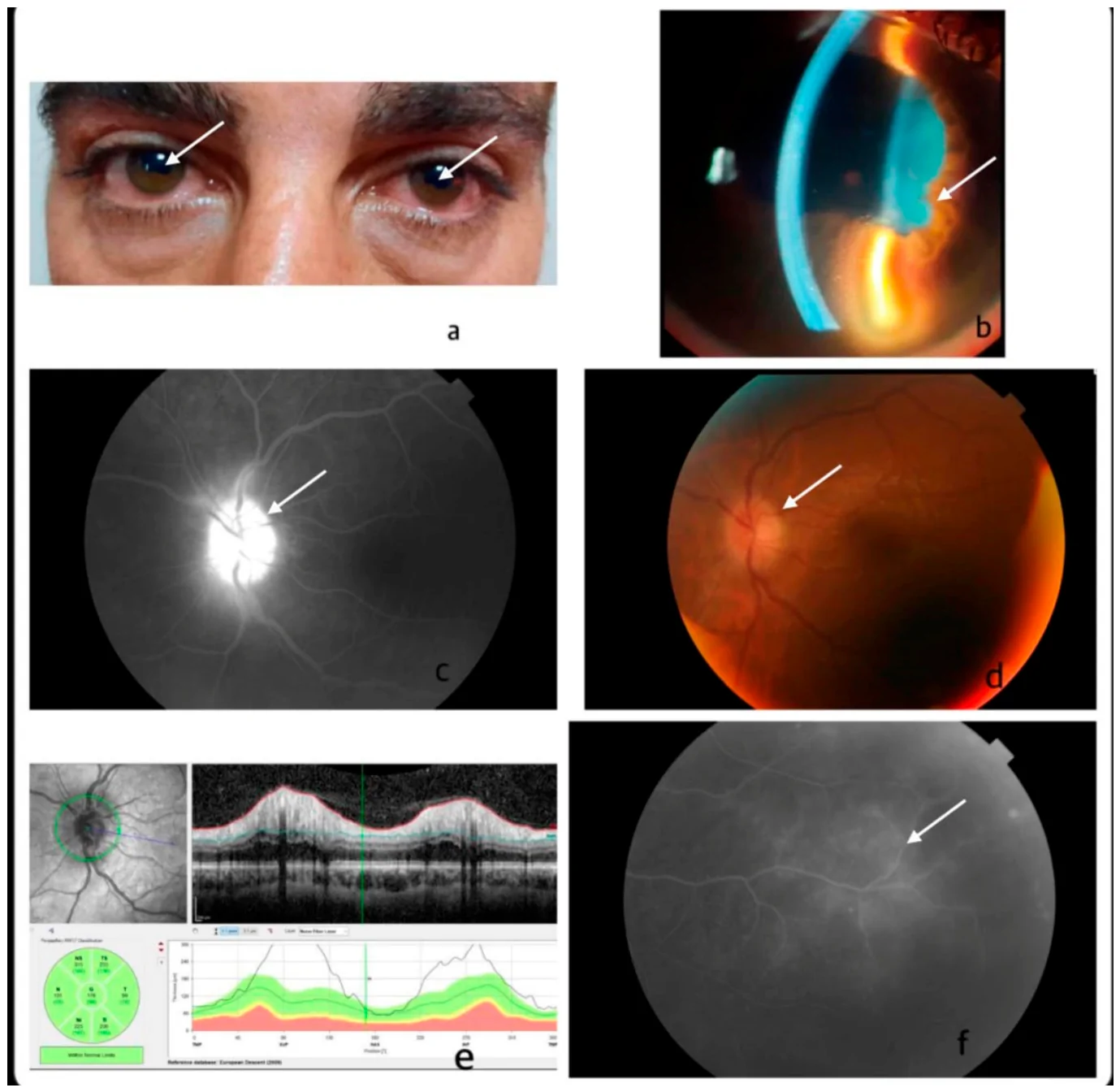
Representative multimodal imaging findings from the same patient with ocular syphilis. (**a**). External examination revealed bilateral conjunctival hyperemia and posterior synechiae (arrow) on pupillary evaluation. (**b**). Slit-lamp examination demonstrated keratic precipitates and posterior synechiae (arrow), consistent with anterior uveitis. (**c**). Fundus fluorescein angiography demonstrated optic disc staining and leakage (arrow), consistent with optic neuritis. (**d**). Color fundus photograph showing optic disc edema (arrow) secondary to optic neuropathy. (**e**). Optical coherence tomography revealed retinal nerve fiber layer thickening, consistent with optic neuritis. (**f**). Fundus fluorescein angiography demonstrated focal retinal vascular leakage (arrow), consistent with retinal vasculitis.

No additional systemic manifestations of syphilis were observed in 14 patients (67%). Among the remaining patients, five presented with maculopapular rashes on the palms, soles, and/or trunk, one exhibited eyelash loss, and one had hair loss involving the eyebrows, eyelashes, and scalp. Representative ophthalmologic findings, including anterior and posterior segment involvement and multimodal imaging features, are illustrated in Figure 1.

### 3.3. Comorbidities and Co-Infections

At least one comorbid condition was present in seven patients (33%). Three patients had both diabetes mellitus (DM) and hypertension (HT), three had DM alone, and one had HT alone. HIV co-infection was identified in four patients (19%), while one patient had hepatitis B co-infection. Intravenous drug use was reported in two patients.

### 3.4. Serologic Findings

Syphilis ELISA was positive in 20 patients. In the patient with a negative ELISA and TPHA, FTA-ABS IgG was positive. Serum VDRL testing was negative in five patients despite positive treponemal serology and positive in 16 patients, with titers ranging from 1:2 to 1:512. TPHA testing was not performed in three patients; however, both ELISA and VDRL tests were positive in these cases.

In all five patients with an initially negative serum VDRL result despite positive syphilis ELISA, VDRL remained negative at the three-month follow-up. Of the 16 patients with a positive VDRL, six did not return for follow-up. Among the remaining patients, serologic responses varied. VDRL became negative in three by the third month. A fourfold decrease in VDRL titers was observed at three months in four patients. In two patients, titers continued to decline until the sixth month, but an increase was noted again at the 12-month follow-up (Table 2). 

### 3.5. Cerebrospinal Fluid Findings and Cranial Imaging

Lumbar puncture (LP) was performed in 14 of the 21 patients (66.6%), regardless of the presence of cranial nerve involvement. The remaining seven patients declined the procedure because of concerns about its potential risks. Of the 14 patients who underwent lumbar puncture, seven had no pleocytosis, whereas the remaining seven had CSF leukocyte counts ranging from 5 to 60 cells/mm^3^. CSF protein levels exceeded 450 mg/L in three patients, while the others were within normal limits. CSF VDRL was positive in two patients, with titers of 1:2 and 1:8.

Regarding cranial imaging, four patients did not undergo cranial imaging. Nine patients underwent cranial CT, and eight underwent cranial MRI. No abnormalities were detected on cranial CT scans. Cranial MRI revealed optic nerve tortuosity in one patient and increased bilateral optic nerve extra-axial CSF distance with bilateral posterior scleral surface scleritis in another.

### 3.6. Patients with HIV Co-Infection

Among the four patients living with HIV, two were female, and the mean age was 44.5 years (33–59). Three patients were newly diagnosed and not receiving antiretroviral therapy (ART) at the time of ocular syphilis diagnosis. ART was initiated concurrently with antisyphilitic treatment in two patients and one week later in the remaining patient. HIV-RNA levels in these patients were 36,070, 38,280, and 43,040 IU/L, with corresponding CD4 T-lymphocyte counts of 260, 166, and 249 cells/mm^3^.

One patient was in the first month of ART at presentation. Although both serum VDRL and TPHA were initially negative, serum VDRL became positive at a titer of 1:64 during the first month of ART. At the time of syphilis diagnosis, this patient’s HIV-RNA was <50 IU/L and CD4 T-lymphocyte count was 387 cells/mm^3^.

Three patients (75%) presented with bilateral panuveitis, while one (25%) had optic neuritis. Vision loss was observed in three patients, whereas one had no ocular complaints. Bilateral ocular involvement was present in three patients. In one patient with nearly complete bilateral vision loss, cerebrospinal fluid analysis revealed 27 cells/mm^3^, and CSF VDRL was positive at a titer of 1:2. Cranial MRI demonstrated bilateral posterior scleral surface scleritis.

### 3.7. Treatment

Regardless of lumbar puncture findings, all patients diagnosed with ocular syphilis received treatment according to the recommended neurosyphilis regimen. Sixteen patients received intravenous crystalline penicillin G (24 million units/day, administered as 4 million units every 4 h), while five patients received intravenous ceftriaxone (2 g/day). The duration of antimicrobial therapy ranged from 10 to 14 days. No Jarisch–Herxheimer reaction was observed during treatment.

Adjunctive systemic prednisolone was administered to 15 of 21 patients (71.4%) according to the severity and anatomical location of ocular involvement. Prednisolone was initiated after the start of antimicrobial therapy on treatment days 1, 7, or 10 at initial doses ranging from 16 to 80 mg/day and was subsequently tapered according to the patient’s clinical response. Patients with anterior uveitis additionally received topical corticosteroids and cycloplegic eye drops. Two patients with posterior segment involvement received posterior sub-Tenon corticosteroid injections while receiving systemic antimicrobial therapy. No patient receiving adjunctive systemic corticosteroid therapy experienced clinical deterioration during follow-up.

Individual patient characteristics, serologic results, cerebrospinal fluid findings, adjunctive corticosteroid use, and treatment regimens are summarized in Table 1.

### 3.8. Visual and Clinical Outcomes

Best-corrected visual acuity (BCVA) was analyzed in 30 eyes of 21 patients. Mean BCVA improved significantly from 0.38 ± 0.30 logMAR (range, 0–1) at baseline to 0.16 ± 0.28 logMAR (range, 0–1) at the final follow-up (*p* < 0.001). No eye experienced deterioration in visual acuity following treatment; BCVA either improved or remained stable in all cases. Furthermore, all cases of uveitis and optic neuropathy achieved clinical remission during follow-up.

## 4. Discussion

The principal finding of the present study is that ocular syphilis remains a diagnostic challenge because negative non-treponemal serologic test results and normal CSF findings do not exclude the diagnosis [6,10]. As delayed diagnosis may result in irreversible visual impairment, early recognition based on a combination of clinical suspicion, ophthalmologic examination, and appropriate serologic testing remains essential [6,11]. With the global resurgence of syphilis, a similar increasing trend has also been observed in Türkiye, atypical manifestations such as ocular involvement are being encountered more frequently in clinical practice [3,4]. Between 2018 and 2023, the number of reported syphilis cases in Türkiye increased substantially [2]. Although a transient decline in reported cases was observed during the COVID-19 pandemic, patients with acute visual symptoms continued to seek medical attention. In our cohort, blurred vision and decreased visual acuity were the most common presenting complaints, likely reflecting the urgency of these symptoms despite healthcare disruptions. This observation emphasizes the importance of maintaining clinical suspicion for ocular syphilis even during periods of healthcare disruption.

The median age of patients with ocular syphilis was 44 (22–78) years, consistent with previous studies and European surveillance data [14,15,16,17], suggesting that the disease predominantly affects young and middle-aged adults. 

The majority of patients were male (87%), and 43% had bilateral involvement. HIV–syphilis coinfection has been reported in approximately 45–60% of patients with syphilis in international studies [12,16,18,19], whereas studies from Türkiye have reported lower rates ranging from 7.6% to 33.3% [14,20,21]. In our cohort, HIV coinfection was identified in 19% of patients with ocular syphilis. However, this proportion should be interpreted with caution because it was derived exclusively from patients with ocular syphilis in a small single-center cohort. These differences are likely attributable to variations in study populations, sample size, referral patterns, and regional epidemiology.

Ocular syphilis most frequently presents with uveitis and optic neuritis, although additional manifestations such as optic disc edema and optic atrophy have also been reported [12,16,22]. In our study, uveitis and optic neuritis were the most common findings, with the majority of uveitis cases (69%) being posterior. While some series report a predominance of posterior or panuveitis, others have highlighted involvement of the anterior segment or optic nerve [9,15]. This variability likely reflects differences in disease stage and the limited sample sizes of most published series, as most available evidence is derived from relatively small case series, limiting definitive conclusions regarding the ocular segments most commonly affected [12,16,23]. It also contributes to the diagnostic challenge of ocular syphilis, as no single ocular manifestation is sufficiently specific to establish the diagnosis. Because of its broad clinical spectrum, ocular syphilis has long been regarded as the “great masquerader,” capable of mimicking numerous infectious and non-infectious causes of ocular inflammation, including herpetic retinitis, cytomegalovirus retinitis, ocular tuberculosis, toxoplasmosis, sarcoidosis, Behçet disease, and other inflammatory uveitic disorders [6,23]. Therefore, ocular syphilis should be considered in the differential diagnosis of unexplained uveitis or optic neuritis, particularly in patients with compatible clinical findings and positive treponemal serology. 

The serological diagnosis of ocular syphilis remains challenging because treponemal and non-treponemal tests provide complementary diagnostic information and should be interpreted together, rather than in isolation [24]. In our cohort, five of 21 patients (23.8%) had a non-reactive baseline VDRL despite positive treponemal testing and ophthalmologic findings consistent with ocular syphilis, consistent with the recent observations of Mohareb et al. that active ocular syphilis may occur despite negative non-treponemal serology [10]. These findings emphasize that a non-reactive non-treponemal test should not reduce clinical suspicion for ocular syphilis when compatible ophthalmologic findings and positive treponemal serology are present. The role of CSF examination in the evaluation of ocular syphilis remains controversial. According to the 2021 CDC Sexually Transmitted Infections Treatment Guidelines, patients with ocular syphilis should be evaluated and treated according to neurosyphilis recommendations regardless of the presence of neurological manifestations [11]. In our cohort, lumbar puncture was performed in 14 of 21 patients (66.7%), yet CSF-VDRL was positive in only one patient, who had HIV infection and presented with severe vision loss. This finding is not unexpected, as CSF-VDRL is highly specific but has limited sensitivity for neurosyphilis [25]. Moreover, half of the patients who underwent lumbar puncture had no CSF pleocytosis, further indicating that ocular syphilis may occur in the absence of CSF inflammatory abnormalities. In our cohort, CSF findings did not influence therapeutic management, as all patients received intravenous neurosyphilis-directed therapy regardless of CSF findings, consistent with current treatment recommendations [11]. Therefore, CSF examination should be considered an adjunct to, rather than a substitute for, clinical assessment, and treatment decisions should be guided by the overall ophthalmologic and serological findings, rather than CSF abnormalities alone [11]. A fourfold decline in non-treponemal titers within 3–6 months is generally considered an adequate serological response following treatment [11]. However, serological responses were heterogeneous in our cohort. While several patients demonstrated the expected decline in VDRL titers or eventual seroreversion during follow-up, others exhibited persistent seroreactivity or fluctuating titers despite receiving standard therapy. Similar variability in post-treatment serological responses has been described previously and does not necessarily indicate treatment failure in the absence of compatible clinical findings. Accordingly, treatment response in ocular syphilis should be assessed by integrating serial serological results with the patient’s clinical and ophthalmologic course, rather than relying solely on serological kinetics.

Magnetic resonance imaging (MRI) may provide supportive information in patients with ocular syphilis, particularly when optic nerve involvement is suspected. Previous studies have shown that MRI findings in syphilitic optic neuropathy are heterogeneous and generally nonspecific, with abnormalities reflecting the site and extent of inflammatory involvement, rather than a characteristic imaging pattern [26]. In our cohort, MRI revealed optic nerve tortuosity, widening of the perioptic cerebrospinal fluid space, and bilateral posterior scleritis in selected patients. Although none of these findings is pathognomonic for ocular syphilis, they were considered supportive of inflammatory involvement of the optic nerve and adjacent ocular structures when interpreted in conjunction with compatible clinical findings, positive serologic results, and the exclusion of alternative infectious and non-infectious etiologies. Therefore, MRI should be regarded as a complementary imaging modality that helps characterize the extent of ocular involvement, rather than a standalone diagnostic tool for ocular syphilis.

Previous studies have shown that HIV coinfection is associated with a higher frequency of panuveitis, posterior segment inflammation, and optic nerve involvement in patients with ocular syphilis [6]. Consistent with these observations, three of four patients (75%) living with HIV in our cohort presented with bilateral panuveitis, whereas one patient had optic neuritis. Visual impairment was observed in three patients, including one who developed near-complete bilateral vision loss. Notably, three of the four patients were newly diagnosed with HIV at the time of ocular syphilis presentation, highlighting the importance of HIV testing in patients presenting with ocular syphilis [6]. Although no conclusions can be drawn from a single case, this observation illustrates the broad clinical spectrum of HIV-associated ocular syphilis. Because only four patients in our cohort were living with HIV, no definitive conclusions regarding the influence of HIV coinfection on the clinical presentation or severity of ocular syphilis can be drawn.

In one patient with HIV/syphilis coinfection, both serum VDRL and TPHA were initially non-reactive; however, VDRL seroconverted to a titer of 1:64 during the first month after initiation of ART. Although the underlying mechanism could not be determined retrospectively, possible explanations include evolving early syphilis that became serologically detectable during follow-up or reinfection during the observation period. Although the prozone phenomenon is a recognized cause of false-negative non-treponemal test results, particularly in patients with secondary syphilis or HIV coinfection, it appears less likely in the present case because both non-treponemal and treponemal tests were initially non-reactive [24]. This observation highlights the complexity of interpreting serological findings in individuals living with HIV and supports repeat serological testing when clinical suspicion for syphilis persists despite initially negative serological results, particularly in patients at ongoing risk for infection [24,27].

Intravenous aqueous crystalline penicillin G (18–24 million units/day) remains the recommended first-line treatment for ocular syphilis according to current neurosyphilis treatment guidelines [11]. In our cohort, ceftriaxone (2 g/day) was administered to five patients (23.8%), primarily because intravenous aqueous crystalline penicillin G was unavailable. Ceftriaxone is considered an effective alternative when intravenous penicillin cannot be used because of its favorable cerebrospinal fluid penetration and convenient once-daily dosing schedule [11].

Adjunctive systemic corticosteroids were administered in 71.4% of patients to control intraocular inflammation. The decision to use adjunctive corticosteroids was individualized according to the severity and anatomical location of ocular involvement. Although high-quality evidence supporting adjunctive corticosteroid therapy in ocular syphilis remains limited, recent reviews and expert recommendations support their selective use after appropriate antimicrobial therapy has been initiated, particularly in patients with severe posterior segment inflammation or optic nerve involvement, with the aim of controlling inflammation and limiting immune-mediated tissue damage, rather than treating the underlying infection [23,28]. No Jarisch–Herxheimer reactions were observed in our cohort during treatment. Likewise, no patient experienced clinical deterioration or worsening of ocular inflammation during therapy, including those who received adjunctive systemic corticosteroids. However, because of the retrospective design, the heterogeneity in corticosteroid use, and the absence of a control group, the independent contribution of adjunctive corticosteroids to clinical or visual outcomes could not be determined. 

This study has several limitations. First, this was a single-center retrospective study with a relatively small sample size, limiting the generalizability of our findings and the statistical power for subgroup analyses. Furthermore, as this study was conducted at a tertiary referral center, more complex or diagnostically challenging cases may have been overrepresented, potentially limiting the applicability of our findings to other clinical settings. Second, incomplete serological follow-up limited the assessment of long-term serological outcomes. Finally, the lack of detailed information on sexual behavior limited the assessment of transmission dynamics. Therefore, our findings should be interpreted as hypothesis-generating and require confirmation in larger prospective studies.

## 5. Conclusions

With the increasing incidence of syphilis, ocular involvement is likely to be encountered more frequently in clinical practice. Our findings highlight that ocular syphilis presents with a broad spectrum of clinical manifestations and variable serological patterns, making timely diagnosis challenging. A non-reactive non-treponemal test or normal cerebrospinal fluid findings should not exclude the diagnosis when compatible ophthalmologic findings and positive treponemal serology are present. Recognition of these diagnostic challenges and adherence to current neurosyphilis treatment recommendations are essential for the appropriate evaluation and management of patients with suspected ocular syphilis.

## Figures and Tables

**Table 1:** Demographic, Clinical, Serological, and Treatment Characteristics of 21 Patients with Ocular Syphilis.

Patient	Age	Sex	HIV	ELISA	Serum VDRL	Serum TPHA	CSF VDRL	CSF Cell Count Cells/mm^3^)	CSF Protein (mg/L)	Symptom	Laterality	Ocular Findings	Treatment (Days)
Patient 1	52	M	-	+	1/32	1/2560	negative	0	314	vision loss	B	posterior uveitis, optic neuritis	Ceftriaxone 2 g/day/10
Patient 2	34	F	-	-	-	-	negative	0	368	photopsia	R	posterior uveitis	Ceftriaxone 2 g/day/14
Patient 3	39	F	+	+	1/128	1/2560	negative	0	223	asymptomatic	B	panuveitis	Ceftriaxone 2 g/day/14
Patient 4	62	M	-	+	1/64	1/2560	negative	0	202	blurred vision	R	posterior uveitis, optic neuritis	Penicillin G 24 MU/day/14
Patient 5	59	M	+	N/A	1/64	1/2560	negative	5	241	blurred vision	B	panuveitis	Penicillin G 24 MU/day/14
Patient 6	47	M	+	+	1/128	1/2560	LP not performed	-	-	blurred vision	L	optic neuritis	Penicillin G 24 MU/day/14
Patient 7	44	M	-	+	1/2	1/640	LP not performed	-	-	asymptomatic	B	posterior uveitis	Penicillin G 24 MU/day/14
Patient 8	78	M	-	+	-	1/1280	negative	0	498	vision loss	B	optic neuritis	Penicillin G 24 MU/day/14
Patient 9	76	M	-	+	-	N/A	LP not performed	-	-	burning and stinging sensation	L	optic neuritis	Penicillin G 24 MU/day/14
Patient 10	40	M	-	+	1/64	N/A	LP not performed	-	-	blurred vision	L	posterior uveitis	Penicillin G 24 MU/day/14
Patient 11	58	M	-	+	1/512	1/640	1/8		322	blurred vision	L	anterior uveitis	Penicillin G 24 MU/day/14
Patient 12	22	M	-	+	-	1/160	negative	60	286	vision loss	B	posterior uveitis	Penicillin G 24 MU/day/14
Patient 13	63	M	-	+	-	1/2560	LP not performed	-	-	blurred vision	B	N/A	Penicillin G 24 MU/day/14
Patient 14	30	M	-	+	1/32	1/2560	negative	0	294	vision loss	R	posterior uveitis	Ceftriaxone 2 g/day/10
Patient 15	41	M	-	+	1/8	1/2560	LP not performed	-	-	vision loss	B	posterior uveitis	Penicillin G 24 MU/day/14
Patient 16	38	M	-	+	1/32	N/A	negative	20	-	vision loss	L	posterior uveitis	Ceftriaxone 2 g/day/14
Patient 17	43	M	-	+	1/32	1/2560	LP not performed	-	-	blurred vision	R	N/A	Penicillin G 24 MU/day/14
Patient 18	63	M	-	+	1/64	1/2560	negative	43	521	vision loss	R	optic neuritis	Penicillin G 24 MU/day/14
Patient 19	23	M	-	+	1/64	1/2560	negative	33	396	blurred vision	L	optic neuritis	Penicillin G 24 MU/day/14
Patient 20	65	M	-	-	1/2	1/2560	negative	2	661	vision loss	R	optic neuritis	Penicillin G 24 MU/day/14
Patient 21	33	F	+	+	>1/256	>1/2560	1/2	27	361	vision loss	B	panuveitis	Penicillin G 24 MU/day/14

CSF: cerebrospinal fluid, VDRL: venereal disease research laboratory, TPHA: Treponema pallidum hemagglutination assay, M: male, F: female, LP: lumbar puncture, B: bilateral, R: right, L: left, N/A: not available, (+) positive, (-) negative.

**Table 2:** Changes in VDRL Titers Following Treatment in Ocular Syphilis Patients.

	Baseline	Month 3	Month 6	Month 12
Patient 1	1/128	1/8	negative	negative
Patient 2	negative	-	-	-
Patient 3	1/128	1/32	1/8	1/32
Patient 4	1/64	negative	-	-
Patient 5	1/64	1/8	-	negative
Patient 6	1/128	1/32	-	1/128
Patient 7	1/2	negative	-	negative
Patient 8	negative	-	-	-
Patient 9	negative	-	-	-
Patient 10	1/64	-	1/2	-
Patient 11	1/512	-	-	negative
Patient 12	negative	-	-	negative
Patient 13	negative	-	-	-
Patient 14	1/32	-	-	-
Patient 15	1/8	-	-	-
Patient 16	1/32	-	-	-
Patient 17	1/32	-	-	-
Patient 18	1/64	-	-	-
Patient 19	1/64	1/32	1/4	negative
Patient 20	1/2	-	-	-
Patient 21	>1/256	1/64	1/16	1/8

Note: (-) indicates that the test was not performed at that time point.

## Data Availability

Data are contained within the article.
